# The XVIII Congress of European Mycologists: Conference Report

**DOI:** 10.3390/jof5040110

**Published:** 2019-11-25

**Authors:** Julia Pawłowska, Magdalena Frąc, Izabela Kałucka, Małgorzata Ruszkiewicz-Michalska, Sylwia Różalska, Marta Wrzosek

**Affiliations:** 1 Department of Molecular Phylogenetics and Evolution, Institute of Botany, Faculty of Biology, University of Warsaw, Al. Ujazdowskie 4, 00-478 Warsaw, Poland; martawrzosek@gmail.com; 2 Polish Mycological Society; Al. Ujazowskie 4, 00-478 Warsaw, Poland; 3 Institute of Agrophysics, Polish Academy of Sciences, Doświadczalna 4, 20-290 Lublin, Poland; m.frac@ipan.lublin.pl; 4 Department of Algology and Mycology, Faculty of Biology and Environmental Protection, University of Lodz, Banacha 12/16, 90-237 Lodz, Poland; izabela.kalucka@biol.uni.lodz.pl; 5 Institute for Agricultural and Forest Environment, Polish Academy of Sciences, Bukowska 19, 60-809 Poznan, Poland; malgorzata.ruszkiewicz@isrl.poznan.pl; 6 Department of Industrial Microbiology and Biotechnology, Faculty of Biology and Environmental Protection, University of Lodz, Banacha 12/16, 90-237 Lodz, Poland; sylwia.rozalska@biol.uni.lodz.pl

The 18th Congress of European Mycologists took place from 16 to 21 September 2019 in Warsaw and Białowieża, Poland (Figure 1). A total of 273 participants from 52 countries presented 77 talks and 176 posters in two offered and 10 thematic sessions: “From genome to function” (led by Ekaterina Shelest, Germany), “Taxonomy and systematics” (led by Jos Houbraken, Netherlands), “Fungi in biotechnology” (led by Katarzyna Turnau, Poland), “Fungal interactions” (led by Martin Bidartondo, United Kingdom), “Medical mycology” (led by Michaela Lackner, Austria), “Fungal diversity” (led by Carrie Andrew, Sweden), “Fungi in primeval forests and other natural habitats” (led by Anders Dahlberg, Sweden), “Hypogeous mycorrhizal fungi” (led by Giovanni Pacioni, Italy), “Fungal conservation” (led by Susana C. Gonçalves, Portugal), and “Data session” (led by Dmitry Schigel, Denmark). The keynote lectures were given by Annegret Kohler (France), David Hawksworth (United Kingdom), Geoffrey Gadd (United Kingdom), Duur K. Aanen (Netherlands), Dominik Begerow (Germany), Marc-André Selosse (France), Bogdan Jaroszewicz (Poland), and Lynne Boddy (United Kingdom). Additionally, two workshops were organized in the Białowieża part of the Congress: “Global fungal red-listing” led by David Minter (United Kingdom) and “Biology of polypores” by Dmitry Schigel (Denmark). An open European Council for the Conservation of Fungi (ECCF) discussion forum entitled „Fungal conservation across borders in Europe (and beyond) for the next four 4 years—what role for the ECCF?” was also held. 

As the motto of the meeting was “Fungi in Nature and Culture”, several congress related mycological exhibitions and events were organized, including a photo exhibition entitled “Fungi—master sculptures of nature”, “Dystopia”—a spatial composition by Hélène Soulier and Ewa Rudnicka, “Anomalium”—a graphics exhibition by Agnieszka Zdziabek, “Broken links”—a performance by Maria Subczyńska, two fresh mushroom exhibitions in Warsaw and an exhibition of the fungi of the Białowieża Forest in Białowieża. On Tuesday evening (17 September 2019), the open lecture “Mycology: a recent weapon in the forensic armoury” was held in the Copernicus Science Centre by Patricia Wiltshire (United Kingdom). Additionally, during the welcome reception, the bench in memory of Prof. Alina Skirgiełło, a distinguished Polish mycologist, was ceremoniously unveiled in the Botanical Garden of the University of Warsaw.

On Tuesday, during the general meeting of the European Mycological Association (EMA), a new board was elected, with Izabela Kałucka (Poland) as President, Mitko Karadelev (Macedonia) as Vice-President, Katerina Rusevska (Macedonia) as Secretary, Eske De Crop (Belgium) as Treasurer, Vijai Kumar Gupta (Estonia) as Membership Secretary, Tatiana Semenova-Nelsen (USA) as Publicity and Social Media Officer, Paulo de Oliveira (Portugal) as EMA Webmaster, and Susana C. Gonçalves (Portugal) as Conservation Officer and Chair of the European Council for the Conservation of Fungi. The outgoing president, David Minter, was warmly thanked for all he had done for the EMA during his tenure. 

During the closing ceremony, it was announced that the 19th Congress of European Mycologists will be organized in Turin, Italy in 2023.

## Figures and Tables

**Figure 1:**
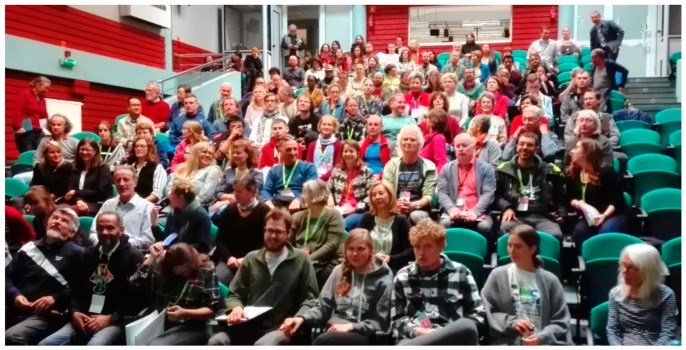
Participants of the 18th Congress of European Mycologists in Białowieża (photo Julia Pawłowska).

